# Chromatin Profiling Techniques: Exploring the Chromatin Environment and Its Contributions to Complex Traits

**DOI:** 10.3390/ijms22147612

**Published:** 2021-07-16

**Authors:** Anjali Chawla, Corina Nagy, Gustavo Turecki

**Affiliations:** 1 Integrated Program in Neuroscience, McGill University, 845 Sherbrooke St W, Montreal, QC H3A 0G4, Canada; anjali.chawla@mail.mcgill.ca; 2 McGill Group for Suicide Studies, Department of Psychiatry, Douglas Mental Health University Institute, McGill University, 6875 LaSalle Blvd, Verdun, QC H4H 1R3, Canada; corina.nagy@mcgill.ca; 3 Genome Quebec Innovation Centre, Department of Human Genetics, McGill University, 845 Sherbrooke St W, Montreal, QC H3A 0G4, Canada

**Keywords:** complex traits, complex diseases, brain, non-coding, epigenome, DNA structure, open chromatin, transcription factors, histone modifications, chromatin loops

## Abstract

The genetic architecture of complex traits is multifactorial. Genome-wide association studies (GWASs) have identified risk loci for complex traits and diseases that are disproportionately located at the non-coding regions of the genome. On the other hand, we have just begun to understand the regulatory roles of the non-coding genome, making it challenging to precisely interpret the functions of non-coding variants associated with complex diseases. Additionally, the epigenome plays an active role in mediating cellular responses to fluctuations of sensory or environmental stimuli. However, it remains unclear how exactly non-coding elements associate with epigenetic modifications to regulate gene expression changes and mediate phenotypic outcomes. Therefore, finer interrogations of the human epigenomic landscape in associating with non-coding variants are warranted. Recently, chromatin-profiling techniques have vastly improved our understanding of the numerous functions mediated by the epigenome and DNA structure. Here, we review various chromatin-profiling techniques, such as assays of chromatin accessibility, nucleosome distribution, histone modifications, and chromatin topology, and discuss their applications in unraveling the brain epigenome and etiology of complex traits at tissue homogenate and single-cell resolution. These techniques have elucidated compositional and structural organizing principles of the chromatin environment. Taken together, we believe that high-resolution epigenomic and DNA structure profiling will be one of the best ways to elucidate how non-coding genetic variations impact complex diseases, ultimately allowing us to pinpoint cell-type targets with therapeutic potential.

## 1. Introduction

Complex traits or diseases are considered to be influenced by interactions between environmental stimuli and regulation of multiple genes. Indeed, correlating allelic frequencies with complex trait variations through case-control genome-wide association studies made it abundantly clear that etiological dissection of complex diseases is non-trivial, and complex diseases are pleiotropic and polygenic. [1,2,3]. The etiological complexity of complex traits can be further influenced by the purging of large effect-size disease-mutations via negative selection, especially those present in the coding-regions. Effectively, this can result in small effect-size variants spread across hundreds of functionally-less deterministic regions [4]. Notably, more than 90% of genome-wide significant risk loci are located in the non-coding regions of the genome, which does not produce proteins, rendering their biological roles elusive [1,2,3,4,5,6]. Large-scale initiatives, such as ENCODE (Encyclopedia of DNA Elements) and REC (Roadmap Epigenomic Consortium), systematically catalogued non-coding elements, providing evidence that at least 80% of the genome is indeed functional [5,6]. As such, non-coding risk loci pose a significant challenge in their functional interpretation or in prioritizing causal variants. This is thought to be partly because of very small effect-sizes of putative risk variants and an insufficient statistical power in pinpointing the causal single nucleotide polymorphisms (SNPs) [1,2,3,4]. Therefore, understanding the regulatory roles of the epi/genome remains a priority.

In addition, the epi/genome can be influenced by the environment factors [7,8,9]. In general, changes in our lifestyle, diet, or social cues, can influence adaptive physiological responses and inter-individual variations in gene expression through epigenetic changes. Moreover, phenotypic heterogeneity in complex traits and diseases point towards the impact of private environment-epigenetic interactions [1,2,3,7]. These unique impacts may also lead to epigenetic variations or de novo mutations precipitated by environmental factors. Indeed, long-term epigenetic and transcriptomic changes have been reported in the brain cell-types of individuals with early-life adversity [8,9]. Therefore, we need improved approaches to identify the combined effects of genetic perturbations and environmental exposures in mediating predisposition to complex traits and diseases.

The epigenomic elements can be broadly defined by regions of open chromatin including cis-regulatory elements, such as insulators, promoters, enhancers, and trans-regulatory binding sites for transcription factors (TFs), as well as histone modification marks that orchestrate a regulatory ensemble, under a dynamic chromatin topology, capable of modulating the transcriptome without altering the nucleotide sequences per se. In turn, the chromatin states and structures are largely influenced by heritable, but also reversible, chemical modifications to DNA and histones, collectively referred to as epigenetic modifications [6,7]. The epigenetic and gene expression changes together regulate cell fate decisions during neurodevelopment. Thereby, the inherent cell-type and epigenetic heterogeneity makes it harder to tease-apart precise molecular modifications and masks subtle disease-related changes when investigating tissue homogenates. Indeed, cell-type proportions were found to be a major contributor to gene expression variations in studies employing bulk tissue homogenates [10]. Hence, single-cell investigations are now increasingly employed over tissue homogenates, to delineate cell-type specific epigenetic programs. Although epigenetic mechanisms like DNA methylation are known to influence gene expression, in this review, we focus on chromatin structure and the respective profiling techniques, including chromatin accessibility, histone modifications, and chromatin topology (Table 1), outlining their applications in deciphering the intricate architecture of complex traits and diseases. To our knowledge, this is the most comprehensive review summarizing these techniques, including state-of-the-art approaches to apply them at single-cell resolution in the brain.

### The Chromatin Environment

**Figure 1:**
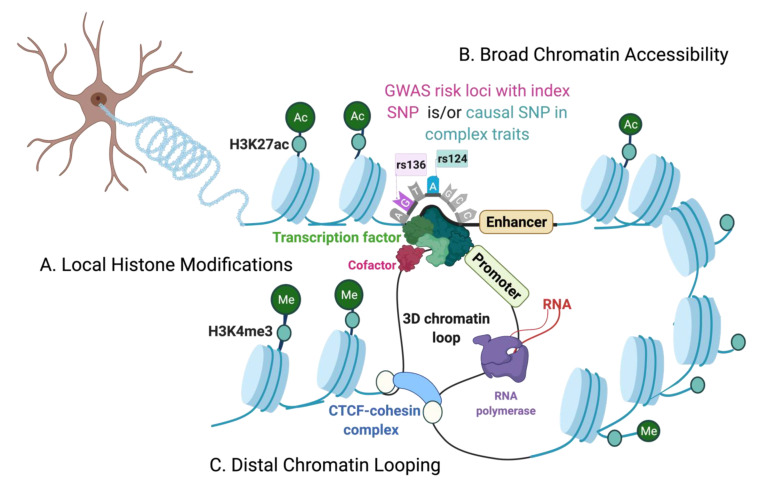
The Chromatin Environment. The open chromatin includes both coding and non-coding aspects of the genome. The interactions between cis- and trans- non-coding, regulatory elements and genes can occur at different genomic scales: locally (such as by histone modifications) or distally (such as by 3-dimesional interactions). The dynamics and functions of the chromatin environment can be mapped using chromatin profiling techniques. (**A**) Local histone modifications (such as acetylation or methylation): induce changes in the chromatin permissiveness, allowing binding of regulatory proteins like transcription factors, impacting expression of the nearby genes. The binding of transcription factors and histone modifications can be assayed using ChIP-seq, CUT&RUN, or CUT&TAG. (**B**) Broad chromatin accessibility: involve significant remodeling of the chromatin landscapes and redistribution of multi-nucleosomes that can directly or indirectly impact expression of multiple genes in the neighborhood. The chromatin environment, cis-regulatory elements and nucleosome distribution can be assayed using ATAC-seq, MNase-seq, DNase-seq, or FAIRE-seq. Genome-wide association studies (GWAS) risk loci for complex traits also largely map to the open non-coding genome, where the index or lead single-nucleotide polymorphism (statistically most significant SNP at a risk loci) may or may not be the disease causative SNP. Identifying regulatory roles of the epigenomic elements associating with risk variants can ascertain causal epi/genetic mechanisms of the complex traits. (**C**) Distal chromatin looping: facilitates long-range gene regulation by DNA elements located farther apart from gene promoters (more than 1–2 kbps), involving 3D changes in the chromatin topology. The spatially interacting genomic regions can be mapped using 3C, 4C, 5C, or HI-C. Additionally, genome-wide chromatin looping interactions of a regulatory protein can be assayed by ChIA-PET, 3C-ChIP, HiChIP, or PLAC-seq.

The chromatin is structurally and functionally active (Figure 1). The two major structural categories of chromatin, associating with distinct histone modifications, include the open regions of chromatin that associate with active gene regulatory mechanisms, collectively called euchromatin, while the nucleosome-dense heterochromatin states are important for defining transcriptionally-inactive regions. A nucleosome is the basic unit of chromatin; a histone protein octamer that wraps ~147bp of DNA, repeated periodically throughout the genome. The accessibility of chromatin (unwound open chromatin) and/or nucleosome positioning at genomic loci are indicative of their regulatory potential, and can be examined using chromatin accessibility techniques, such as DNase-seq (DNase I hypersensitive sites sequencing) and MNase-seq (micrococcal nuclease digestion of chromatin followed by sequencing) (Figure 1B). Typically, active regulatory regions are thought to be depleted of nucleosomes to allow RNA polymerases or TFs to bind in mediating gene expression. Additionally, the DNA around nucleosomes can transiently unwrap to allow regulatory factors to bind, known as “DNA breathing” [11].

More stable nucleosome post-translational modifications are facilitated by ATP-dependent chromatin remodeling complexes, such as SWI/SNF (Switch/Sucrose non-fermentable) and nucleosome remodeling and deacetylase complex (NuRD). More commonly, histone-remodeling enzymes, such as histone acetyl- or methyl- transferases, can lead to covalent modifications at the N-terminal tails or core of the histone proteins [12]. The activity of histone remodeling enzymes in repositioning nucleosomes at chromatin regions can be regulated by the availability of metabolic cofactors. For example, histone acetyltransferases (HAT) depend on acetyl-CoA to neutralize positive charge of lysine-rich histone tails by adding an acetyl group, destabilizing electrostatic interactions with the DNA, and opening the local chromatin. In contrast, the histone deacetylases (HDAC) are dependent on the availability of Zn^2+^ or NAD^+^ cofactors to remove the acetyl groups, restabilizing the chromatin structure [12]. Thereby, histone modifications regulating changes in the chromatin environment are conducive to the binding of transcriptional repressor or activators and can be assessed by ChIP-seq (chromatin immunoprecipitation with sequencing) (Figure 1A) and alternative techniques (Table 1).

In general, histone modification patterns at regulatory sites, such as at promoters, can effect local chromatin permissiveness to TFs in regulating proximal gene activity, while large-scale histone modifications and nucleosome redistribution either directly or indirectly leading to the remodeling of chromatin accessibility landscapes can impact long-range gene regulation, and can be assessed by ATAC-seq (assay for transposase-accessible chromatin coupled to sequencing) or ChIA-PET (chromatin interaction analysis by paired-end tag sequencing). Moreover, these interactions can be reversible (e.g., to maintain cellular functions) or stable to define cell lineages (e.g., during neurodevelopment) [7].

The non-coding elements commonly effect distal gene expression through 3-dimensional (3D) chromatin interactions or loops, involving shifts in the chromatin topology. Chromatin loops spatially juxtapose functional loci and gene promoters to facilitate long-distance gene expression or insulate genomic regions with diverse chromatin states. These higher-order chromatin interactions can be mapped by chromatin conformation techniques, such as the 3C or Hi-C (Figure 1C). 

Of note, the CCCTC-binding factor (CTCF) is a transcription factor that colocalize with ring-shaped cohesin complexes to organize the formation of 3D chromatin loops (Figure 1C), as well as the topologically associated domains (TADs). TADs are structural units comprising genomic regions with high interaction frequencies. Additionally, the CTCF-cohesin complexes also act as transcriptional insulators, blocking enhancer-promoter interactions, and repressing gene expression. Importantly, genetic mutations in the CTCF complexes are linked to neurodevelopmental delays [13]. Overall, the chromatin-profiling techniques for assaying distinct epigenetic features are thoroughly compared and reviewed (Table 1).

**Table 1:** Comparison of chromatin profiling techniques to assaying epigenomic features.

Epigenomic Features	Techniques	Methods Overview	Benefits	Limitations	Single-Cell and Cell-Types
1. Open chromatin regions. 2. Cis-regulatory elements.	DNase I hypersensitive sites sequencing (DNase-seq). [14]	DNase I digested fragments are extracted using biotin-streptavidin complex.	1. High signal-to-noise ratio compared to FAIRE-seq. 2. No prior knowledge of locus-specific sequences, primers, or epitope tags is required. 3. Efficiently maps non-coding regions proximal to genes.	1. DNase I sequence-specific cleavage biases may determine cleavage patterns at the predicted transcription factor (TF) binding sites or footprints. This complicates correctly assessing true transcription factor binding at open chromatin. [15] 2. Requires high number of cells (ideally >= 1 M cells) [14] and a high sequencing depth. 3. Maps relatively low distal regulatory sites compared to formaldehyde-assisted isolation of regulatory elements with sequencing (FAIRE-seq). [16]	Single-cell (sc)-DNase-seq. [17]
1. Nucleosome positioning. 2. DNA-bound protein binding sites.	Micrococcal nuclease digestion of chromatin followed by sequencing (MNase-seq) [18], (alternative: nucleosome occupancy and methylome sequencing (NOME-seq). [19]	Cross-linking to covalently link proteins to the DNA, followed by micrococcal nuclease digestion to remove free DNA.	1. MNase-seq can map DNA-protein binding for both histone and non-histone proteins. 2. Indirectly maps chromatin accessibility. 3. The digested fraction of accessible chromatin can be repurposed for chromatin immunoprecipitation-based assays (Native-ChIP).	1. Requires a broad range of sequencing read-out (25 bps to 150 bps) to capture both sub-nucleosome and nucleosome fragments. [20] 2. High dependency on optimized MNase enzyme digestion for reproducibility between experiments. 3. MNase enzyme produces AT cleavage bias that needs bioinformatic corrections. 4. Requires large number of cellular input (ideally >= 1 M cells).	scMNase-seq, and scNOME-seq. [21,22,23,24]
1. Open chromatin. 2. Cis-regulatory elements. 3. Nucleosome distribution.	Assay for transposase-accessible chromatin coupled to sequencing (ATAC-seq). [25]	Tn5 transposases-based cutting and tagging of open chromatin.	1. Low input (ideally <= 50,000 cells) 2. Short and easy to use protocol. 3. Very high signal-to-noise ratio compared to other chromatin accessibility techniques.	1. Tn5 sequence insertion bias can lead to mapping and/or TF footprinting biases and needs bioinformatic corrections. 2. Mitochondrial contamination of reads (although Omni-ATAC [26] is optimized for lower mitochondrial reads).	Flow cytometry-based approaches and single cell/nucleus ATAC-seq. [27,28,29,30,31,32]
1. Protein-DNA interactions. 2. Histone post-translational modification.	Chromatin immunoprecipitation with sequencing (ChIP-seq). [33,34,35]	Formaldehyde crosslinked (X-ChIP) or micrococcal digested fragments (Native-ChIP) followed by immunoprecipitation.	1. Gold standard to map genome-wide, direct DNA-protein interactions. 2. Single-nucleotide resolution (compared to ChIP-qPCR and ChIP-chip). 3. An ultra-low-input micrococcal nuclease-based native ChIP (ULI-NChIP) can profile genome-wide binding sites of histone proteins with as few as 1000 cells. [36]	1. Cross-linking and sonication steps (X-ChIP) can lead to high background noise, requiring higher cellular input for optimal signal-to-noise ratio. [33] 2. Relies on the availability and quality of specific antibodies and can suffer from epitope masking due to cross-linking of fragments (X-ChIP). 3. Requires appropriate control experiments to minimize detection of false-positive protein-DNA binding sites.	sc-ChIP-seq [37]
1. Protein-DNA interactions. 2. Histone post-translational modification.	ChIP with exonuclease (ChIP-exo) [38], Cleavage under targets & release using nuclease (CUT&RUN) [39], Cleavage under targets and tagmentation. (CUT&TAG) [40]	ChIP-exo: X-ChIP immunoprecipitated fragments followed by additional λ exonuclease digestion step. CUT&RUN: MNase tethered protein A, targeting specific antibody against the protein of interest. CUT&TAG: Tn5 transposase and protein A fusion protein, targeting antibody against the protein of interest.	1.ChIP-exo: with an extra exonuclease treatment, it can remove unbound and non-specific DNA, providing higher signal-to-noise ratio over ChIP-seq. [38] 2. CUT&RUN: (i) Uses enzyme-tethering to avoid cross-linking and fragmentation of DNA that greatly reduces the background noise, and epitope masking, making it lower input over ChIP. (ii) It has been validated to map H3K27me3-marked heterochromatin regions. [39] (iii)Use of enzyme-tethering also maps local environment of binding sites, making it suitable to also detect long-range interactions of the protein. 3. CUT&TAG: (i) Requires the least number of cells compared to alternatives (ideally >= 100 cells) and can be performed at single-cell level. [40] (ii) It bypasses cross-linking (compared to ChIP) and library preparation step (compared to ChIP and CUT&RUN). (iii) More sensitive, easier workflow and cost-effective compared to CUT&RUN and alternatives	1. ChIP-exo: High number of enzymatic steps in ChIP-exo makes it technically challenging and suffers from epitope masking, similar to ChIP. 2.CUT&RUN: (i) Calcium-activated MNase enzyme digestion of chromatin needs to be carefully optimized, to prevent over/under digestion of accessible chromatin. It also relies on antibody quality, like ChIP. (ii) Like X-ChIP, CUT&RUN cannot distinguish direct from indirect 3D contacts. [39] (iii) Requires higher number of cells relative to CUT&TAG (ideally >= 100,000 but can be performed with as low as 1000 cells). [39] 3. CUT&TAG: (i) A potential limitation is antibody-validation, since mapping certain protein-DNA interactions can be more efficient after cross-linking. (ii) Tn5 enzyme biases may confound detection of proteins at heterochromatin regions, since Tn5 preferentially tags accessible chromatin	CUT&TAG [40]
3. Chromatin loops and 3D interactions.	Chromosome Conformation Capture 3C [41], 4C [42], 5C [43], and Hi-C. [44]	Formaldehyde cross-linking to covalently link physically interacting chromatin fragments.	3C/4C/5C: these progressive modifications can map increasingly more chromatin conformations, i.e., one-to-one, one-to-many, and many-to-many epigenetic features, respectively. Hi-C (all-to-all): 1. An unbiased approach that maps genome-wide 3D chromatin conformations. 2. Long-range interactions several mega-base pairs away and high-resolution inter-chromosomal contacts can also be mapped. 3. Low cellular input over 3C/4C (ideally >= 1 M cells). Easy-Hi-C: a biotin-free strategy, more sensitive and requires relatively lower cell input over Hi-C (ideally >= 50,000 cells). [45]	3C/4C/5C: 1. Maps to a limited resolution and genomic distances of interacting regions. 2. Need priori-defined regions of interests. 3. Cannot resolve long-range contacts by haplotypes (maternal/paternal) of the chromosomes. 4. Requires relatively higher number of cells (ideally >= 10M cells). Hi-C: (i) It cannot detect chromatin contacts with cell-type specificity and cannot detect functional relevance of the chromatin loops. (ii) Some proximity-ligation events can remain undetected due to low efficiency of biotin incorporation at ligation junctions. [45]	Flow cytometry-based approaches [46,47], sc-Hi-C-seq [48,49], sci-Hi-C-seq [50,51], Dip-C [52]
4. Protein-bound 3D interactions	Chromatin interaction analysis by paired-end tag sequencing (ChIA-PET) [53], HiChIP [54], and Proximity ligation-assisted ChIP-seq (PLAC-seq). [55]	Formaldehyde cross-linking, followed by antibody-based immunoprecipitation of protein-bound chromatin interactions.	ChIA-PET, HiChIP & PLAC-seq: Can illustrate regulatory roles of 3D chromatin interactions. HiChIP & PLAC-seq: Higher signal-to-noise ratio and significantly lower cell input compared to ChIA-PET.	ChIA-PET: 1. Low sensitivity in detecting 3D interactions and can have false-positive reads by non-specific antibody binding. 2. Requires very high number of cellular input (ideally >= 100 M cells) [54,56] and high sequencing depth. 3. Ligation of DNA linkers to chromatin fragments can also lead to self-ligation of linkers and false-positive read-outs. ChIA-PET, HiChIP, and PLAC-seq: They all require a priori of target protein of interest and need bioinformatic correction for biases introduced by: ChIP procedure, different fragment lengths, and restriction enzymes cut-site biases. HiChIP and PLAC-seq also require high cell-number (ideally >= 1 M cells).	Flow cytometry approach [55,57], and multiplex chromatin interaction analysis via droplet-based and barcode-linked sequencing (ChIA-Drop) [58]

## 2. Chromatin Accessibility Techniques

Regions of open chromatin include coding and non-coding aspects of the genome. Interestingly, they harbor the majority of the genome-wide significant risk variants associated with neuropsychiatric disorders [1,2,3], and they are subject to remodeling by neuronal plasticity and therapeutic drugs [59,60]. A number of gene regulatory mechanisms can be investigated through the following techniques. 

### 2.1. DNase I Hypersensitive Sites Sequencing (DNase-seq)

DNase-seq leverages the DNase I enzyme that digests only the open chromatin regions, and not the nucleosome-packed inactive heterochromatin, generating DNase I hypersensitive sites (DHSs). These sites encompass cis-regulatory elements, locus control regions, and transcription factor binding sites, allowing identification of functional non-coding elements. Optimal DNase I digestion is carried out to enrich for the nucleosome-free regions from the isolated nuclei. To reduce random shearing, DNase I digested DNA is embedded in low-melt gel agarose plugs, followed by synthesis of blunt ends. The extracted chromatin is ligated to biotinylated linkers for subsequent enrichment of small DNA fragments using streptavidin columns, followed by PCR amplification and hybridization to microarrays (DNase-Chip) [61] or high-throughput sequencing (DNase-seq) [14].

DNase-based high-throughput analyses of open chromatin have been widely employed to investigate regulatory functions of the non-coding regions and non-coding disease risk loci [5,62,63]. ENCODE initiatives mapped and characterized about 3 million unique DHSs using DNase-seq across hundreds of cell-types. While this represented on an average 1% genome in each cell type, it covered more than 90% ENCODE-identified binding sites of transcription factors [5]. Complex trait and disease risk variants catalogued by the National Human Genome Research Institute (NHGRI), were found to overlap strongly with ENCODE DHSs (34%), the majority of which overlapped with functional enhancers and/or the TSSs. Moreover, up to 71% of complex traits associated SNPs were found to be likely functionally causative in DHSs when those in the linkage disequilibrium (LD; alleles that are non-randomly associated within a population) were included, among which 31% directly overlapped TF binding sites [5]. This demonstrated that the majority of risk SNPs associated with complex traits and diseases could potentially impact regulatory functions of the non-coding elements. 

Likewise, collectively employing multiple databases such as ENCODE, REC, and fetal DHSs, resulted in the association of thousands of noncoding SNPs to functional DHS sites, either directly or in LD (76%), for hundreds of complex diseases, and reproducibly, 93% of DHS SNPs overlapped TF binding sites. The candidate DHSs harboring disease risk variants were among those that mediated changes in chromatin accessibility and associated with distal gene promoters. The associations of gene promoter with DHSs were based on the significant correlations (Pearson correlation coefficient > 0.7) in their DNase I hypersensitivity signals within 500 kbps radius. This further suggested that functional DHSs that were found to be associated with complex disease risk variants could regulate distal gene promoters [63]. Taken together, these studies described an approach to identify causative SNPs at non-coding regions, whose functions otherwise are not easily understood. 

Since the disruption of TF binding sites is considered to be an important mechanism by which non-coding variants mediate disease pathogenesis [5,63], many techniques have been developed for characterizing their binding to the genome. Transcription factor footprinting [64] is one such approach that can predict TF occupancy due to the relative changes in DNase cleavage events created by bound TFs along the genome, generating the resulting footprints. Employing this technique across 29 brain-tissue samples showed that TF binding sites contributed disproportionately to the heritability of brain-related traits and psychiatric diseases. Further, the TFs associated to those sites were found to be enriched for neurodevelopmentally-related functions. However, brain TF footprints were found to more variable across test samples compared to other tissue types [64], likely indicating higher cell-type heterogeneity. Therefore, future studies accounting for cellular complexity should reveal deeper insights into precise regulatory mechanisms. 

Although footprinting approaches rely on the ability of TF bound sites to be more resistant to cleavage by DNase digestion, accumulating evidence suggests that TFs with shorter DNA residence time leave minimal footprints [15], illustrating a correlation between TF binding kinetics and footprinting depth. Thereby, footprinting predictions can be factor-dependent and should be carefully interpreted at dynamic timescales.

Human-specific DHSs were defined as regions with human-specific increase in DNase-seq signal compared to non-human primates. These DHSs were shown to be cell-type specific (present largely in one cell-type) and primarily enriched at distal enhancers [65]. Notably, species-specific changes to chromatin accessibility correlated with species-specific differences in gene expression and recognition sequences of TFs, such as for activator protein-1 (AP-1), a key activity-dependent TF that modulates synaptic plasticity [65]. Moreover, brain-specific DHSs that show evidence of accelerated evolution (brain-aceDHSs) were enriched for target genes with differential expression between humans and chimpanzees [66]. These brain-aceDHSs also overlapped several human-specific TF motifs, including CTCF and early growth response 1 (EGR1) motifs, important for chromatin organization and activity-dependent functions. Importantly, putative risk SNPs associated with complex traits and brain diseases also overlapped with brain-aceDHSs [66]. Taken together, these studies suggest that at least some gene-regulatory elements at open chromatin landscapes are under adaptive evolution, including those that are fundamental to neurodevelopment and cognition. Further, these regions may also confer risk to neuropsychiatric diseases through unfavorable epi/genetic variations.

A stratified LD score regression can be employed to estimate contributions of functional epigenetic elements to heritability of complex traits. Using this approach, active DHSs were shown to explain higher proportions of complex trait heritability compared to coding regions [67]. Moreover, heritability enrichments for complex traits were cell-type specific, for example, enrichment for psychiatric traits were specific to brain tissues and cell-types that overlapped histone marks associated with open chromatin and functional enhancers. These findings highlight the importance of studying tissue- and cell-specific epigenetic elements in dissecting disease etiology. 

To examine cell-type specific differences in epigenomic signatures, a large number of biological replicates are required as produced by ENCODE; however, this may not be feasible for the primary tissues. Furthermore, deconvolution approaches require specific epigenetic markers for distinct cell-types, which remain approximative at best. More sensitive approaches that can allow unbiased cell-type specific investigations are inclusive of single-cell investigations.

Single-cell DNase sequencing (scDNase-seq) has been shown to generate cell-type specific DHSs. Briefly, this method involves flow cytometry based single-cell sorting, DNase I digestion, and addition of circular carrier DNA to minimize loss of digested short fragments, followed by preferential amplification of small DNA fragments and sequencing [17]. This method detected 38 thousand DHSs per cell, and was sufficient to identify cell-type specific enhancers regulating gene expression programs. Further, this approach was successfully implemented to identify complex disease mutations at regulatory regions effecting target gene expression in specific cell-types [17]. As such, scDNase-seq can be used to identify novel cis-regulatory elements or causal risk SNPs underlying disease phenotypes with cell-type specificity and future work should consider implementing this technique. 

### 2.2. Formaldehyde-Assisted Isolation of Regulatory Elements with Sequencing (FAIRE-seq)

FAIRE-seq, like DNase-seq, maps open regions of the chromatin. It relies on crosslinking protein bound chromatin with formaldehyde followed by nuclei isolation and lysis, sonication, and reversal of cross-links to obtain 200–1000 bp fragments. Finally, phenol-chloroform extraction can separate the organic phase containing unused covalently-linked protein complexes, from the aqueous phase with protein-free DNA. The isolated DNA can subsequently be paired with quantitative amplification (qPCR), hybridized to microarrays, or libraries can be prepared for high-throughput sequencing [16]. 

A combination of DNase-seq and FAIRE-seq in human cell lines encompassed 9% of human genome across cell-types and captured significantly more TF binding sites than either technique by itself. Despite the mostly overlapping nucleosome-free regions between the two techniques, there is a degree of uniqueness to each approach. FAIRE-seq captured more distal regulatory sites enriched in H3K4me1 histone marks, while DNase-seq captured open regions more proximal to TSSs enriched in H3K4me3 and H3K9ac histone marks. Together, these complementary approaches resulted in a higher-resolution mapping of cis-regulatory elements. Interestingly, open chromatin regions shared across cell lines were generally proximal to TSSs and enriched for CTCF binding sites. On the other hand, open chromatin associated with specific cell types was relatively depleted of CTCF binding sites but enriched for major cell-type defining TFs thought to coordinate cell-type specific gene expression [68]. Therefore, combining profiles of open chromatin regions from these two techniques provides deeper insight into human regulatory epigenome.

The differential properties of the FAIRE-seq and DNase-seq in mapping cis-regulatory elements are likely the result of technical differences. These include distinct regulatory proteins bound at the open chromatin regions that could impact formaldehyde cross-linking in FAIRE-seq. Likewise, relative depletion of nucleosomes proximally to genes may be more susceptible to DNase I digestion [68].

Given the accumulating evidence suggesting that risk SNPs in complex diseases are often located farther from gene bodies [64], FAIRE-seq is useful for probing distal enhancer loci. For example, FAIRE-seq-identified cis-regulatory elements in a patient-based cohort showed that the germline and somatic variants of complex diseases correlated with disruption in TF binding sites at differentially accessible enhancer regions and their accompanied altered gene expression [69]. In addition, these approaches could ascertain clinical sub-categories of the disease. FAIRE-seq combined with ATAC-seq was also used to identify key TFs that regulated distinct stages of disease progression through chromatin remodeling, whereby a loss-of-function mutation in a key disease-related TF decreased severity of the disease [70]. FAIRE-seq is not as widely implemented, possibly due to its inability in determining open chromatin regions bound to regulatory proteins (TF/RNAPII), as a result of formaldehyde cross-linking of DNA-bound proteins. Despite this, FAIRE-seq offers certain advantages, such as circumventing the requirement of an enzymatic step or nuclei suspensions, and can be paired with other chromatin techniques for investigating larger epigenomic landscapes [71]. 

### 2.3. Micrococcal Nuclease Digestion of Chromatin Followed by Sequencing (MNase-seq)

One of the most popular methods to determine nucleosome occupancy is MNase-seq. Other similar methods include nucleosome occupancy and methylome sequencing (NOME-seq) that map nucleosome position along with DNA methylation [19] or site-directed chemical cleavage of nucleosomes [72]. MNase-seq employs an endo-exonuclease called the micrococcal nuclease, isolated from *Staphylococcus aureus*, which digests linker DNA and accessible chromatin between nucleosomes, without degrading the nucleosomes. A typical MNase-seq protocol involves crosslinking chromatin with formaldehyde to prevent digestion of histone bound DNA, nuclei isolation, micrococcal digestion to remove free DNA. Subsequently, cross-linking is reversed, and proteinase K digestion is used to release histone proteins. DNA is extracted with phenol-chloroform or spin columns and used as input for microarrays [73], or high-throughput sequencing [18,20]. 

Employing MNase-seq in human cell lines showed that nucleosome occupancy is dependent on distinct DNA methylation and histone modification patterns [74]. For example, H3K4me3-histone marks, associated with active promoters, were generally depleted of nucleosomes, while H3K9me3-marked inactive epigenetic elements had relatively higher nucleosome occupancy [74]. On the other hand, distinct nucleosome distribution at TF binding sites can determine lineage-specific TFs. An increased nucleosome occupancy at binding sites of Stat3 and p300 TFs was found in the lineage-committed cells compared to embryonic stem cells and neural progenitor cells (NPCs) [75]. Interestingly, combining ENCODE ChIP-seq and MNase-seq datasets led to the development of an unsupervised chromatin pattern discovery tool that predicted asymmetry and heterogeneity in distribution of nucleosomes and histone modifications flanking distinct classes of TF binding sites [76]. 

In general, and on an average across cell-types, most eukaryotic chromatin has a nucleosome repeat length of 185–195 bp, corresponding to ~147 bp of nucleosome DNA and ~45 bp of linker DNA. However, nucleosome spacing can also be indicative of specific cell-types and/or disease-states. For example, MNase-seq in distinct cell-types identified a shorter average nucleosome spacing in dorsal root ganglia neurons (~165 bp) compared to cortical astrocytes or oligodendrocyte precursor cells (~183 bp) [77]. Another study depicted age-dependent effects on nucleosome spacing and reported that nucleosome spacing on an average increased with age (up to 50 bp) in mammalian cortical and cerebellar neurons, but not in the glial cell-types [78]. As such, epigenetic changes (such as DNA methylation) have been shown to correlate with ageing process [9]. Given that precise nucleosome spacing at regulatory sites is an important determinant of transcriptome, it will be important to test, whether and to what extent, age-dependent changes in the neuronal epigenome relate to age-related changes in synaptic functions.

MNase-TSSs sequence capture is a modified technique to map nucleosome distribution surrounding only TSSs at a genome-wide scale. This approach identified nucleosome relocation around TSSs at early stages of the disease. This, in turn, was associated with aberrantly high TF binding and disruption of gene expression programs that mediate disease progression [79]. Moreover, alterations to nucleosome occupancy around gene TSSs has been associated with both neurological [80] and psychiatric diseases [81]. Chromatin remodelers can increase nucleosome density, displacing RNAPII and leading to gene silencing [82]. Moreover, mutations in chromatin remodelers have been reproducibly associated with neurodevelopmental and psychiatric disorders [82,83]. Taken together, nucleosome turnover by chromatin remodeling factors can impact interactions at cis-regulatory elements, dysregulating target gene expression. 

Combining human *de novo* mutation datasets with MNase-seq-derived nucleosome maps revealed that non-coding regions at/around translationally stable nucleosome positioning across cell-types associate with significantly higher de novo mutation rates, INDELs, repeat elements, and a lower DNA replication fidelity of those sites [84]. This further suggests that nucleosome positioning may be an important factor in determining DNA mutation rate variations, which associate with numerous complex traits and diseases.

Recently, single-cell MNase-seq has been able to obtain nucleosome positioning and chromatin accessibility profiles from single cells [21]. Briefly, fluorescence assisted cell (FAC)-sorting of single cells can be paired with native or fixed cells and micrococcal nuclease digestion of single-cell or bulk cell suspension can be carried out depending on the amount of starting material, followed by phenol-chloroform extraction of DNA fragments. Isolated DNA is ligated with specific adapters for PCR amplifications and subsequently purified for high-throughput sequencing [21]. This approach revealed nucleosome organizing principles of cell-types, not evident in bulk MNase-seq. For example, smaller variations in the positioning of nucleosomes were detected within single cells and cell-types than those found across different cell-types. Furthermore, scMNase-seq demonstrated that the nucleosomes surrounding both the active DHSs and transcription start sites of active genes showed less positional variance across different cell-types and correlated with variations in gene expression, as compared to inactive DHSs or silenced genes [22].

Other single-cell methods include scNOMe-seq that can measure both nucleosome occupancy and DNA methylation at a genome-wide scale [23]. Multi-omics approaches, such as scNMT-seq (single-cell nucleosome, methylation and transcription sequencing), can directly identify impacts of nucleosome positioning on transcriptomic regulation at the single cell level [24]. These techniques have allowed us to integrate different but complementary levels of genomic information, providing multimodal signatures for a given cell.

### 2.4. Assay for Transposase-Accessible Chromatin (ATAC-seq)

ATAC-seq can capture multi-nucleosome regions of open chromatin using at least 10 times less nuclei and can obtain a higher signal-to-noise ratio compared to the previously described DNase, FAIRE, or MNase-seq. Introduced by Buenrsotro et.al, ATAC-seq requires a prokaryotic Tn5 transposase charged with point mutations to increase its enzymatic activity and adaptors to tag accessible chromatin. Tn5 transposase is applied to the isolated nuclei in bulk. Specific primer pairs can be used to amplify the cut and tagged segments of DNA, which is then followed by high-throughput sequencing. A successful ATAC-seq library shows a laddering pattern with 200 bp periodicity, corresponding to segments of DNA devoid of one (200 bp) or more nucleosomes [25]. With slight modifications, such as the use of multiple detergents and post-lysis nuclei washing with Tween-20, Omni-ATAC-seq is optimized for long-term frozen tissues and attains lower mitochondrial contamination. The use of this adapted protocol with postmortem brain tissue showed enrichments for neurological and psychiatric disease associated risk variants in regions of open chromatin [26].

ATAC-seq has become a popular technique for studying DNA structure, not only because of its ease of use, but also because of its robust findings. For example, the Common Mind Consortium (CMC)-led study in postmortem human brain identified about 9% SNP heritability in schizophrenia in the open regions of chromatin. In addition, a four-fold increase in the SNP heritability for this illness was found when including evolutionarily conserved open regions [85]. Interestingly, differences in accessibility across open regulatory regions appear to be significantly influenced by age and disease phenotypes. Cellular maturation influences the closing of regulatory loci enriched for motifs important for activity-dependent dendritic patterning and NPCs self-renewal. Schizophrenia-related phenotypic alterations were correlated with changes in open chromatin enriched in motifs important for neurogenesis and myelin regeneration [85]. Furthermore, many quantitative trait loci (QTLs) that were found to impact chromatin accessibility changes in the brains of individuals with schizophrenia, showed concordant effects with QTLs effecting gene expression changes (eQTLs), suggesting an association of specific alleles and chromatin states with gene expression alterations in diseased phenotypes. Of note, this study used a very large sample-size, but did not correct for cell-type heterogeneity in chromatin states [85].

Since ATAC-seq can be performed on small amounts of material, researchers have successfully used fluorescence-activated nuclei sorting (FANS) to isolate broad cell types based on antibodies against specific cell markers. Generating neuronal (NeuN+) and non-neuronal (NeuN-) populations from postmortem brain regions of healthy individuals showed that individual cell-types capture more than 50% of the variance in open chromatin brain regions, in contrast to biological sex that accounted for less than 2% variance [27]. Additionally, the neuronal open chromatin showed less overlap with the bulk DHSs than non-neuronal cells, potentially indicating higher variability among neuronal subtypes. Moreover, open chromatin regions of neurons were mostly distal and intergenic with more variable profiles across brain regions than non-neuronal open chromatin [27], suggesting region-specific distal gene regulation in neurons.

Overlapping risk loci with open chromatin regions revealed that neurons from the striatum and hippocampus were enriched for schizophrenia risk variants, while non-neuronal hippocampal regions were enriched for risk variants associated with major depressive disorder (MDD) [27]. Likewise, an organoid model of forebrain development (cell sorted by FACS) depicted both time- and lineage-specific accessibility patterns that correlated with distal enhancer accessibility (+/− 500 kbps of TSSs) of glial and neuronal marker gene expression. In terms of disease association, schizophrenia-associated risk variants were enriched across mature neuronal or non-neuronal cell-types, while those for autism spectrum disorders were enriched primarily in progenitor glial cells [28], further highlighting the importance of employing cell-type specific modalities.

Combining ATAC-seq with a more refined FANS approach by sorting for glutamatergic neurons, GABAergic neurons, oligodendrocytes, and microglia/astrocytes resulted in cell-type specific differentially open coding- and noncoding-regions [29]. For example, differentially open chromatin overlapping *Bdnf* gene was found in the glutamatergic neurons, while open chromatin of *Lhx6* gene was detected in the GABAergic neurons. In addition, cell-type specific open chromatin overlapped with regulatory regions of cell-type specific marker genes. Further, TF footprinting using ATAC-seq, such as DNase-seq, can predict binding of TFs at open chromatin. The footprinted TFs were associated with target genes by the distance of TF binding sites to TSSs. Moreover, the target genes of cell-type specific TFs were among those with cell-type specific open chromatin [29]. These results elucidate the role of accessible chromatin in influencing cellular transcriptome.

The open chromatin regions in glutamatergic neurons showed strong enrichments for risk variants associated with psychiatric phenotypes including schizophrenia and brain-related traits like neuroticism and intelligence [29]. Moreover, cell-type deconvolution of bulk ATAC-seq from the brains of individuals with schizophrenia [85] using cell-type open chromatin signatures identified in this study, further implicated glutamatergic cell-type in pathology of schizophrenia [29]. On the other hand, microglia/astrocytes cell types were enriched for Alzheimer’s disease (AD) risk related SNPs. Together, these findings support the need to acquire cell-specific epigenome when investigating complex phenotypes [29].

Single-cell or nucleus ATAC-sequencing (sc/sn-ATAC-seq) can capture cells that cannot be isolated through gene markers (i.e., FANS based isolation), as well as identify landscapes of rare cell-types and/or cell-states. Using the principles of bulk ATAC-seq, scATAC-seq requires a fluidics-based chip, where single cells are captured into individual wells, followed by Tn5 transposition and amplification. Single-cells are then barcoded for cell-identification, and subsequently pooled for library generation and next-generation sequencing (NGS) [30]. Alternatively, a high-throughput droplet-based sequencing can be done using 10x chromium microfluidics, where cells are transposed in bulk, and then isolated with a gel bead matrix so every region of open chromatin from a given cell is tagged with a unique 16 bp cell specific barcode sequence. This approach was used to profile distinct regions of the developing human forebrain, revealing regulatory mechanisms essential for neurogenesis with cell-type and cell-state specific chromatin landscapes and those associating with germline and de novo disease risk variants of complex psychiatric traits [31].

A plate-based combinatorial barcoding approach called sci-ATAC-seq was established to allow multiplexing of high numbers of cells/nuclei. First, one-to-few nuclei are tagged with barcoded Tn5 in a single well of a 96-well plate, and then it is followed by a fixed number of successive barcoding events with different barcode and pools of nuclei, enabling multiplexing of cells, making it scalable and cost-efficient [32]. This approach was used to develop an atlas of 45 distinct brain regions from the adult mice, identifying almost 492,000 cis-regulatory elements, which could define 160 cell-type clusters [86]. The majority of the cis-regulatory elements (96%) were located at least 1kbp away from promoter regions. Among 1% of invariant cis-regulatory elements across the cell-types, 80% were at promoters and others mainly at CTCF binding sites. The open chromatin from mice leveraged with coordinates converted to human genome, revealed significant overlaps of complex brain disease risk variants with open chromatin regions with both regional and cell-type specificity [86].

The use of bulk-ATAC-seq captured minimal enrichments for Alzheimer’s or Parkinson’s disease associated risk variants, however, combining it with snATAC-seq revealed five-fold enrichment of SNPs overlaying regions of open chromatin at cell-type specific regulatory loci [87]. Further, SNP heritability for Alzheimer’s and Parkinson’s were mainly predicted to occur in microglial cells. Both microglia-specific TF binding sites and gene targets were found to be enriched for risk SNPs, while heritability for other neurological or psychiatric traits were mostly predicted in distinct neuronal cell-types [87]. These findings strongly point towards the importance of using single-cell techniques when studying complex disorders of the brain.

Taken together, the general patterns of chromatin accessibility and disease enrichments consistently show distal regulation of cell-type specific genes. Risk variants for psychosis-associated diseases are mainly enriched in the open regions of neurons, while neurodegenerative disease variants occur more consistently in open chromatin regions of non-neuronal cell-types. These findings hold true across distinct chromatin accessibility measuring approaches [88,89,90].

## 3. Chromatin-Bound Proteins and Histone Modifications

Mounting evidence suggests that histone-remodeling factors mediate open/closed chromatin states, which in-turn alters the binding of TFs and other cofactors in mediating gene expression. Moreover, these histone remodelers are capable of keeping regulatory regions in a stable configuration over time. The modification can even be maintained after passage of the replication fork, thereby, sustaining a long-term “epigenetic memory” over cell generations and preserving cell- or lineage-specific gene expression programs [91].

### 3.1. Chromatin Immunoprecipitation with Sequencing (ChIP-seq)

Chromatin immunoprecipitation or ChIP is a widely employed technique for assaying protein-DNA interactions by using specific antibodies [33,34,35]. This is often paired with microarrays technology (ChIP-chip) or high-throughput sequencing (ChIP-seq) for high throughput analysis or with qPCR for site-specific interrogations. Typically, ChIP protocols employ cells that are treated with formaldehyde to cross-link proteins of interest to the chromatin (e.g., TFs or RNAPII) followed by sonication called the X-ChIP. Alternatively, cells are digested with MNase enzyme (without cross-linking) to enrich for DNA associated with nucleosomes to probe for histone modifications (this method is referred to as Native-ChIP). These are followed by immunoprecipitation of the protein-bound DNA with specific antibodies, reversing the cross-links (in case of X-ChIP), and size-selecting DNA to generate libraries for sequencing [33].

ChIP-seq has been used extensively to map important functional, non-coding regions of the genome, by either defining histone modifications to a chromatin state or mapping various transcription factors to genomic regions. In a seminal 2007 ChIP-seq study, genome-wide binding sites of a repressor element-1 silencing transcription factor (REST) were identified in the human T cell lines [34]. In this study, REST was found to be a negative regulator of neuronal gene expression in non-neuronal cells. Around the same time, Native-ChIP in T cells revealed correlations of histone modification patterns with gene activity, for example, H3K4me1, H3K9me1, and H2A.Z variants were associated with both functional enhancers and promoters. On the other hand, promoters were additionally associated with higher H3K27me1 or H3K9me1 signals downstream of transcription start sites [35].

ChIP-seq in neuroblastoma cell lines was used to probe genome-wide binding sites of TCF4, a transcription factor known to regulate the excitability of cortical pyramidal cells while its dysregulation has been associated with numerous cognitive deficits [92]. This study revealed that TCF4 recognition sites contain E-box sequences and H3K27ac histone mark for active enhancers. Interestingly, nearly half of all schizophrenia risk loci identified by the psychiatric genomic consortium contained a TCF4 binding site. Further, TCF4 binding sites were detected near genes important for neurodevelopment and genes harboring de novo mutations for neuropsychiatric disorders. Thereby, this ChIP-seq study elucidated regulatory mechanisms of TCF4 transcription factor in associating with psychiatric disorders [92].

Super-enhancers are defined as broad stretches of multiple enhancers spanning open chromatin regions and strongly associated with histone acetylation signals. In addition, the super-enhancers are often found to be associated with a transcriptional coactivator, Med1. Typically, super-enhancers allow binding of cell-type specific TFs and regulation of cell-type specific gene expression [93]. Of note, although certain disease-associated motifs are enriched at regions considered as super-enhancers, they are not completely recognized in the field as an independent regulatory entity. Nonetheless, cell-type specific approaches, such as FANS of postmortem cortical neurons from individuals diagnosed with schizophrenia, showed differential H3K4me3 ChIP-seq signals at numerous loci compared to controls. H3K4 hypermethylated regions were highly enriched at super-enhancers containing myocyte enhancer factor 2C (MEF2C) motifs, crucial for synaptic regulation. Interestingly, multiple MEF2C motifs were also found within schizophrenia risk loci, while *Mef2c* overexpression in cortical neurons of adult mice improved cognitive performance after psychotogenic drug treatment [94].

H3K4me3 ChIP-seq in human prefrontal cortical neurons, compared to chimps and macaques, revealed hundreds of human-specific methylation gains that correlated with dysregulation of genes implicated in psychiatric disorders, including *CACNA1C*, *ADCYAP1*, *DPP10*. Interestingly, increase in human-specific H3K4 methylation at the 5′ promoter of psychiatric-risk gene, *DPP10*, correlated with its downregulation via transcription of an antisense RNA [95]. Therefore, H3K4me3 signal-gains correlate with open chromatin but can also associate with gene activity and negatively regulate gene expression at neurodevelopmentally-important genes [95]. Interestingly, open regions with human-specific gains in methylation in neurons showed increased human-specific sequence alterations, including SNPs and INDELs, not present in the neighboring coding-regions [95]. These findings suggest that human-specific genetic changes can play a role in defining human-specific histone methylation status in the regulatory genome. Furthermore, the regulatory loci harboring hominid footprints include those that confer psychiatric risk, whereby unfavorable epigenetic changes may increase susceptibility to psychiatric diseases.

H3K4me3 ChIP-seq in neuronal and non-neuronal cells from prenatal, young, and elderly human prefrontal cortex revealed cell-type specific dynamic remodeling from mid-gestational to early postnatal life (up to 2 years postnatally) but only minimal changes from adolescence to adulthood. Developmentally regulated H3K4me3-signals were within 2 kbps of age-related and synaptic genes. In addition, developmentally upregulated H3K4me3 peaks in cortical neurons were enriched for activity-dependent AP-1 motifs [96]. Likewise, the neuronal epigenome of normal infants showed an excess of H3K4me3-signals at several neurodevelopmentally-important gene promoters compared to older brains. Moreover, developmentally-regulated peaks mapped mostly within 2 kbps of gene TSSs, while, subject-specific H3K4me3 peaks were largely distally located (more than 10 kbp from TSSs) [97]. Overall, these studies illustrated age-dependent reorganization of neuronal epigenome in a cell-type and subject-specific manner.

Rapid epigenetic changes specific to early-life at activity-dependent gene regulators [97] support the possibility of an early cortical remodeling window vulnerable to environmental perturbations. In addition, numerous studies have identified epigenetic variations at early-developmental periods disrupting transcriptome in complex neurodevelopmental disorders [98,99,100].

Although ChIP-seq from tissue homogenates has highlighted potential disease-related targets, the lack of cell-type features can mask subtle histone-modifications, which might be driven by cellular composition rather than phenotype. As such, we have witnessed the development of single-cell ChIP-seq approaches using drop-seq based microfluidics and barcode multiplexing. Of note, cell-types clustered accurately based on H3K4me3 or H3K27me3 histone marks associated with permissive or repressive transcription [37]. Indeed, the specificity of scChIP-seq is immediately obvious when investigating patient-derived breast cancer xenografts that had acquired resistance to therapy. A subset of cells within therapy-sensitive tumors lost the repressive H3K27me3 signals at gene loci involved in therapy-resistance, similar to the resistant tumors [37], indicating sustained epigenetic modifications resulting in altered transcriptional responses in specific cell-types, undetectable via bulk approaches.

### 3.2. ChIP-seq Alternatives

#### 3.2.1. DNA Adenine Methyltransferase (DAM)-Identification (DamID)

ChIP-seq has some limitations, such as nonspecific DNA binding or uneven fragmentation that can contaminate immunoprecipitants leading to spurious or false-positive reads. Another limitation of X-ChIP is the requirement of sonication that can cause high background noise, necessitating higher cellular input for optimal signal-to-noise ratio [33]. Recently, modifications to the Native-ChIP protocol requiring significantly lower cell input (as low as 1000 cells) by FAC-sorting cells directly into nuclei lysis buffer has been described [36]. Further, an adaption of Native-ChIP to profile non-histone proteins in low-salt conditions to preserve protein-DNA interactions has also been described [101]. Although, cross-linking protein-DNA interactions can be useful to avoid redistribution of highly dynamic TFs, several antibodies can be limited in their applicability to cross-linked fragments due to epitope masking. This has motivated the use of alternative methodologies, such as enzyme-tethering to non-fixed cells in DamID.

This technique uses *Escherichia coli* Dam tethered to protein-of-interest, which can catalyze N^6^-methylation of adenines in GATC sequences present in their vicinity. The methylated regions are digested with *DpnI*, followed by microarray-hybridization or high-throughput sequencing [102]. DamID-seq has been used to reveal transcription factor binding sites using minimal number of cells and generate high-density gene regulatory networks for TFs, such as POU5F1 and SOX2, among others, previously implicated in several psychiatric disorders [103].

Targeted DamID in embryonic mouse cortex characterizing CHD8 binding sites, a chromatin remodeler with de novo mutation associated with sporadic autism spectrum disorder, indicated that binding of CHD8 at distal enhancers regulates neurodevelopment-associated genes, e.g., *ANK3* [104]. Labeling specific genes with Dam can generate models that can be used to map early-life epigenetic modifications that can mediate long-term susceptibility to psychiatric diseases [105].

Single-cell DamID in human cell lines provided a “molecular contact memory” approach that was used to map fates of regulatory loci spatially interacting with nuclear lamina over time. This was also found to correlate with H3K9me2 histone marks for transcriptional repression [106]. Furthermore, scDam&T (transcriptome)-seq, a multi-omics approach, has allowed direct correlations of regulatory protein-DNA contacts with mRNA changes in a cell-type specific manner [107]. A key limitation of DamID is that it is biased to GATC locus, and generally requires transgenic cells [108].

#### 3.2.2. Cleavage under Targets and Release Using Nuclease (CUT&RUN)

The drawbacks of ChIP-seq and DamID have motivated development of alternative methodologies, including CUT&RUN. Briefly, unfixed nuclei are immobilized on magnetic beads and incubated with MNase-tethered staphylococcal protein A (pA-MN) that binds to antibodies targeting protein-of-interest. This is followed by Ca2+ ion treatment for induction of double-stranded DNA cleavage and centrifugation to separate protein-bound DNA for sequencing, generating long-range protein-interactions maps at single-bp resolution [39].

The early postnatal maturation of the brain follows a precise epigenomic remodeling, and environment-prompted alterations in these stages are associated with increased susceptibility to diseases [105]. CUT&RUN was used to identify the postnatal switches regulating brain maturation by probing for multiple transcription factors [109]. Methyl CpG binding protein 2 (MECP2), a transcriptional repressor, showed selective binding at embryonic enhancers in cortical neurons of adult mice, partly dependent on postnatal de novo CG methylation at embryonic enhancers. Moreover, a significant increase in CUT&RUN H3K27ac signals (enhancer-specific) was observed in the *Mecp2* conditional knockout mouse cortex. Thereby, site-specific methylation and MECP2 binding were found to be important mechanisms regulating postnatal long-term decommissioning of neuronal enhancers [109]. On the other hand, the activity-dependent FOS displayed increased binding at postnatally activated neuronal enhancers. In other words, specific neuronal subtypes showed de novo enhancer enrichment and associated with newly expressed genes postnatally. Footprinting of FOS CUT&RUN regions revealed enrichment for activity-dependent AP-1 motifs, while mutations in AP-1 motifs decreased H3K27ac-signals at postnatally activated enhancers, delineating their importance as postnatal switches. Notably, postnatal changes in H3K27ac-enriched distal enhancers strongly correlated with postnatal gene expression changes.

Additionally, CUT&RUN for ARID1A, a subunit of SWI/SNF BAF chromatin remodeling complex, showed increased binding at FOS-bound postnatally induced enhancers by 3 weeks postnatally. Further, the binding of ARID1A at postnatally activated enhancers continued into the adulthood, which likely maintained them in an active configuration through nucleosome repositioning [109]. The culmination of these findings suggests that postnatal switches regulate early-life decommissioning of embryonic enhancers along with activity-dependent activation of postnatal enhancers. These epigenetic mechanisms are maintained into adulthood and are important for postnatal brain development and functions. Notably, mutations in the subunits of SWI/SNF complexes, disrupting chromatin states, have been associated with numerous neurodevelopmental and psychiatric disorders [83]. Therefore, CUT&RUN can be advantageous in determining genome-wide binding sites of regulatory proteins with low cell input [109], and those with relatively sparse tissue expression, such as the estrogen receptor-α (ER-α) in the brain [110].

An interesting alternative to CUT&RUN is cleavage under targets and tagmentation (CUT&TAG), which is principally the same but employs Tn5 transposase for tagmentation of DNA sequences near the binding sites of a regulatory protein, making it ultra-low-input and more sensitive than CUT&RUN [40]. Briefly, hyperactive Tn5 transposase tethered to protein A fusion protein (pA-Tn5) is charged with sequencing adapters and requires Mg^2+^ ions for Tn5 activation and integration of adapters to protein binding sites, generating chromatin fragments ready for amplification and sequencing [40].

Schizophrenia-associated genes identified from snRNA-seq in postmortem brains were found to be highly regulated by a few TFs (SATB2, SOX5, MEF2C, and TCF4), also overlapping GWAS risk loci [111]. CUT&TAG was used to validate binding regions of these TFs in cortical neuronal nuclei sampled from schizophrenia and control individuals, which showed an overlap of TF target genes with snRNA-identified differentially expressed genes in neuronal sub-types. Mapping active regulatory regions at TF-bound sites revealed functional enrichment patterns for neurodevelopmental and postsynaptic-related functions, two commonly proposed mechanisms for schizophrenia pathogenesis [111]. These results further suggest that the risk for complex disorders can be conferred by disruptions in binding sites of key TFs, leading to alterations in their target gene network, in specific cell-types.

## 4. 3D Chromatin Interactions: Techniques and Applications

In biology, structure follows function, for example, chromosome territories (CT) compartmentalize gene rich and poor regions, and their shuffling has been reported in pathological states [112]. Further, disruption of 3D chromatin interactions at gene regulatory regions can lead to functional consequences. For example, point mutations in the RNAPII associated transcription factors have been found to repel chromatin loop formation between gene promoter-terminator sequences, disinhibiting multiple rounds of transcription, and leading to gene expression changes [113]. Whether these chromatin loops are spatially or temporally disrupted in mediating complex diseases warrants deeper investigations.

### 4.1. Chromosome Conformation Capture

#### 4.1.1. 3C: One-to-One Mapping

Spatial interactions between any two genomic loci can be mapped using 3C, a technique introduced by Dekker et al. in 2002. Briefly, intact nuclei are fixed and cross-linked by formaldehyde resulting in formation of covalent bonds between physically interacting chromosomal segments bridged by proteins. Cross-linked chromatin is digested with restriction enzymes to retain only physically linked fragments, followed by ligation, reversal of cross-links to form chimera of interacting fragments and PCR amplification with locus-specific primers. A control without the ligation step validates physical interactions [41].

The 3C-qPCR in postmortem cortical neurons showed 3D-interacting H3K4me3-peaks up to 1 Mb apart associated with human-specific increases in H3K4 methylation at loci implicated in neurodevelopmental disorders and encompassing psychiatric-risk genes [95]. Therefore, altered and unfavorable 3D interactions overlapping histone methylation changes in the regulatory genome could increase susceptibility to complex disorders.

MHC (major histocompatibility) complexes, central to immune-related functions, have been strongly implicated in psychiatric disorders by GWAS [114], albeit most of the risk variants were located far from gene bodies. Employing 3C in postmortem cortical tissue has revealed multiple 3-dimensional interactions between risk variants at active enhancer regions that mapped to these complexes and distal genes [115,116]. The implication of MHC suggests potential immune-related dysfunctions [117] and points toward the effect of adverse environment-epigenetic interactions in mediating vulnerability to complex diseases.

Of note, ChIP-3C assay involving an antibody-based immunoprecipitation of cross-linked, physically interacting loops illustrates their functional roles. MECP2 ChIP-3C showed that MECP2 binding was necessary for chromatin loop-mediated gene silencing of imprinted genes [118]. The aberrant transcription of silenced genes or loss of imprinting by disruption in MECP2-mediated interactions may be one of the mechanisms in mediating susceptibility to neurodevelopmental disorders [118]. Notably, chromatin loops are necessary for activity-dependent long-range communications between promoter-enhancer regions while their disruption dysregulates gene activity in psychiatric phenotypes [118,119].

#### 4.1.2. 4C: One-to-Many Mapping

Given that a regulatory locus can interact with multiple other loci, for example, an enhancer regulating expression of several genes, one-to-many mapping is particularly informative. The 4C-Chip, also known as 3C-on-Chip, takes the advantage of high-throughput microarray technology paired with the 3C technique [42]. In addition, 4C-seq employing NGS has also been described [120]. Briefly, 3C-ligated templates undergo another around of digestion with a secondary restriction enzyme and are re-ligated to form small DNA circles that can be amplified by inverse PCR, followed by purification of DNA fragments and sequencing [120]. Moreover, 4C circumvents the need for prior knowledge of the interacting loci and can detect both intra and inter-chromosomal interactions. This technique showed that while inactive X chromosome lacked organized looping interactions, escapees like *Xist* were involved in 3D interactions with each other [121]. The 4C-seq has also been widely employed to map gene-regulatory networks interacting with disease-risk loci [122,123,124].

#### 4.1.3. 5C: Many-to-Many Mapping

Chromosome Conformation Capture Carbon Copy, also called 5C, maps interactions among many regulatory loci at the same time. Post-3C cross-linking and ligation, 5C employs a multiplexed ligation-mediated amplification using primers pairs that anneal across 3C-ligated junctions and can be paired with microarray or sequencing. For example, 10,000 5C primers can generate up to 25 million distinct chromatin interactions [43,125].

5C-seq has shown that long-distant spatial configuration disproportionally mediates gene expression in mammalian cells [126]. A study of the impact of neuronal activity on 5C chromatin loop architecture in cortical neurons revealed that activity-dependent gene expression correlated with 3D interaction frequencies of their promoters with distal enhancers marked by H3K27 acetylation [127]. Engagement of activity-induced de novo loops anchored at activity-dependent enhancers significantly increased gene expression, while the overall complexity and size of 3D interactions correlated with temporal expression of activity-dependent genes. Additionally, activity-regulated looped enhancers enriched for risk variants associated with psychiatric disorders. These results indicate that activity-regulated enhancers can impact adaptive gene expression responses to environmental changes. Since dysregulation in activity-dependent signaling has been previously associated with neurodevelopmental disorders, such as the autism-spectrum disorder [128], risk variants at activity-regulated enhancers could increase maladaptive responses and vulnerability to psychiatric traits.

Using 5C-seq to generate a CTCF connectome showed that most gene-enhancer connections anchored by CTCF in pluripotent cells are lost during embryonic differentiation and neural lineage-commitment [129]. As such, depletion of CTCF in postmitotic neurons led to learning and memory loss and disrupted long-range interactions with synapse related genes, while CTCF binding sites are often associated with risk variants for neuropsychiatric diseases [130,131]. Yin Yang 1 (YY1), another major chromatin architect-like CTCF, generally nests within constitutive CTCF frameworks. Knocking down YY1 in NPCs led also to chromatin loop ablation at several enhancer-promoter sites correlating with alterations in expression of neurodevelopmentally-important genes [129]. Together, these studies suggest that aberrations in chromatin architecture and mutations at distal regulatory loci can disrupt long-range gene interactions, while some physical interactions can get permanently lost during neurodevelopment.

#### 4.1.4. Hi-C: All-to-All Mapping

Hi-C provides an unbiased genome-wide mapping of all the genomic loci paired with high throughput sequencing. Briefly, cells are cross-linked with formaldehyde that results in covalent links between 3D-interacting chromatin fragments. Chromatin is digested with restriction enzymes and 5′-overhangs are filled. This is followed by addition of biotinylated residues and ligation, after which biotin-fragments are enriched with streptavidin beads and Hi-C libraries are constructed and sequenced. This technique was used to validate compartmentalization of human genome into A/B sections within the nucleus, where A is open, active, and accessible compared to B [44]. 

Topology maps of human corticogenesis from distinct postmortem frontoparietal regions including cortical plate (comprising postmitotic neurons) and germinal zone (with mitotically active neural progenitors) of mid-gestational fetuses were generated using Hi-C [132]. Integrating the Hi-C interactome with enhancers that had human-specific H3K27ac or H3K4me2 epigenetic gains during cerebral corticogenesis showed approximately 65% of enhancers did not interact with their adjacent genes. Moreover, 40% of genes, involved in regulating human-specific cognitive traits and risk for intellectual disabilities, interacted with enhancers in a region-specific manner. These findings demonstrated the importance of generating tissue- and cell-type specific topological maps. Likewise, Hi-C studies have demonstrated that complex disease risk loci at non-coding regions often influence distal gene expression by engaging 3D interactions in a neural lineage-specific manner [132,133]. Thus, it is important to investigate epigenetic interactions during distinct stages of neurodevelopment in a cell-type specific manner, in addition to measuring end-point differences in the diseased-states.

PsychENCODE is a harmonized collection of transcriptomic, open chromatin, and Hi-C interactome data, across cortical brain regions for 1866 individuals. Altogether, 90,000 enhancer-promoter long-range interactions and cell-type specific 3D interactions within 2735 CTCF-bound TADs have been cataloged. From these data, a distinct pattern was reported for fetal and adult Hi-C connectome. Interestingly, it was found that eQTLs distal to gene promoters supported by Hi-C enhancer-promoter interactions had significantly higher association with gene expression than those eQTLs located within the gene promoter or exons and not supported by Hi-C interactions [134]. Thus, Hi-C interactions are quite informative in associating genetic risk variants with their target genes. Further, by combining eQTLs, transcription factor-gene interactome, and long-range enhancer-promoter interactions with disease risk variants to gene targets, psychiatric phenotypes could be predicted with six-fold higher accuracy compared to using additive polygenic risk scores [134].

Hi-C chromatin maps from 21 adult human tissues identified another major architectural feature called frequently interacting regions (FIREs), with significantly higher cis-connectivity and cell-type specificity. FIREs were detected to be mostly located in compartment A within TADs, and were found to be partly dependent on CTCF-cohesin complex for their formation [135]. Additionally, neurological disease-related SNPs were found enriched at super-enhancers in FIREs within the brain tissue [135].

A low input easy-Hi-C protocol, which improves the resolution of proximity-ligation events through a biotin-free strategy, in situ proximity-ligation, and an extra exonuclease step to remove un-ligated contaminants, was successfully applied to both adult and fetal postmortem human brain tissues and cell lines [45]. Employing the easy-Hi-C topological maps, authors demonstrated that chromatin loops perform better than eQTLs in predicting target genes associated with distal risk loci [45]. Moreover, 3D chromatin contacts have been shown to identify regulatory functions of non-coding risk variants more reliably than paradigms based on LD [136]. Interestingly, easy-Hi-C-seq in postmortem fetal and adult brain cortical tissues also revealed that A/B compartments, tissue-specific FIREs, and chromatin interactome together, are significant and orthogonal predictors of gene expression [137]. In addition, 3D chromatin interactions anchored at functional enhancer/promoter loci connected the highest number of target genes to the risk loci for brain-related traits and psychiatric disorders, as compared to eQTLs and linear gene proximity approaches. Likewise, chromatin loops showed substantial SNP heritability for psychiatric diseases [137]. Together, these findings highlight the advantages of using 3D long-range interactions for identifying risk genes associated with disease risk loci.

Given a striking difference in percentage of gene loops between NPCs, neuronal, or glial cells [133], single-cell Hi-C studies are imperative. Although, Hi-C has been paired with flow cytometry-based sorting of cell-types [46,47], it cannot distinguish cell-to-cell differences in chromatin structure. Introduced in 2013, sc-Hi-C-seq follows bulk Hi-C protocol performed in the intact nuclei. Individual nuclei are subsequently selected using microscopy, and biotinylated Hi-C ligation junctions are purified on streptavidin-coated beads. These purified fragments are ligated with adapters, PCR amplified, followed by multiplexing of cells and library sequencing. This technique validated cell-to-cell variability in chromosome structure and showed that active genes are located preferentially at the boundaries of chromosome territories across all cells [48]. Other alternatives involve combinatorial indexing-based sci-Hi-C-seq [50,51]. Employing a modified sc-Hi-C-seq protocol that allows imaging of single cells before capture in mouse ESCs showed concentric rings of A/B surrounding an internal nucleolus across all cells and a strong correlation between gene expression and locational depth within the A compartment [49].

Single-cell chromatin topology maps using diploid chromatin conformation capture, Dip-C, demonstrated clustering of distinct cell-types based on cell-type specific enhancer-promoter 3D contacts [52]. By eliminating single-cell biotin-pulldown and performing single-cell isolation using flow cytometry followed by whole genome amplification using multiplex end-tagging amplification, authors achieved higher sensitivity in detection of spatially-interacting chromatin regions with minimal false-positive captures [52].

A major challenge in 3D reconstruction of diploid genome is accurately identifying chromosome haplotypes involved in spatial interactions. Since non-coding SNPs disrupting 3D chromatin interactions are identified in complex diseases [118,119], spatial localization of genetic variants is important. Using haplotype-resolved or phased SNPs, authors were able to distinguish the two haplotypes of each chromosomes. This confirmed the allele-specific 3D connectome at the imprinted *H19/IGF2* gene locus [52], and 15q11 or Prader-Willi/Angelman syndrome locus, suggesting that 3D chromatin reorganization may be one of the mechanisms underlying imprinting disorders [138]. Further, Dip-C in mouse cortex and hippocampus across early postnatal to adulthood periods indicated major structural, compositional, and transcriptomic reorganization one month postnatally. These findings were independent of early-life experiences, and occurred at neurodevelopmentally important loci [138]. Taken together, these studies elucidated chromatin reorganization with cell-specificity during neurodevelopment, and 3D organizing principles of the genome.

### 4.2. Protein-Centric 3D Interactions

Although Hi-C can provide genome-wide mapping of chromatin contacts, it cannot provide precise functional roles mediated by chromosomal loops. Similar to 3C-ChIP, chromatin interaction analysis by paired-end tag sequencing (ChIA-PET) allows genome-wide mapping of chromatin interactions mediated by regulatory proteins. Briefly, formaldehyde cross-linked DNA is sonicated and enriched for protein-of-interest with specific antibodies followed by proximity-based ligation with DNA linkers and extraction of 20bp paired-end tags for sequencing [53]. The seminal study in 2009 described extensive estrogen receptor-α bound long-range interactions at numerous gene promoters, supporting coordinated gene expression [53]. ChIA-PET has also been used to map long-range chromatin interactions associated with RNAPII in human cell lines showing that promoter-promoter interactions encompassing multiple genes were transcriptionally coordinated, while enhancer-promoter interactions involving a single gene were generally cell-type specific, developmentally regulated, and included enhancer sites that mapped to disease risk loci [139].

An ENCODE ChIA-PET study showed that cohesin-bound loops were present at a sub-TAD scale and cell-type specific cohesin-loops were enriched for disease risk loci, unlike invariant TAD boundaries across cell-types [140]. Further, resolving allele-based chromatin topology by long-read ChIA-PET showed genetic variants at regulatory sites repelled CTCF binding and loop formation effecting target gene expression in an allele-specific manner [56]. Long-range physical interactions of transcription start sites with distal enhancers was interrogated with RNAPII ChIA-PET interactome. This showed that risk variants at distal enhancers could alter stress-associated transcriptomic responses in conferring psychiatric disease risk [141].

ChIA-PET requires millions of cells and greater read-depth for assaying 3D interactome, precipitating the development of other techniques. HiChIP (Hi-C with ChIP) involves cross-linking and digestion of DNA fragments in the intact nuclei for Hi-C library construction followed by ChIP. This technique generated cohesin-mediated interactome in human cell lines with 100-fold less nuclei input and 10-fold higher read-depth relative to ChIA-PET [54]. H3K27ac targeted HiChIP in postmortem human brain localized hundreds of neurological diseases-associated SNPs at spatially interacting enhancer-promoter loci, identifying candidate risk genes [87].

In addition, long-range chromatin interactions can also be mapped by the proximity ligation-assisted ChIP-Seq (PLAC-seq), principally similar to HiChIP, and is more sensitive than ChIP [55] The majority of H3K4me3 or H3K27ac PLAC-enriched interactions overlapped with active promoters and enhancers, respectively [55]. H3K4me3 PLAC-seq in FAC-sorted cell-types revealed that PLAC-interaction strengths across genomic loci were sufficient to cluster cell-types in the developing human cortex by developmental age and influenced cell-type specific gene expression. Additionally, H3K4me3 PLAC-interacting distal sites associated with risk variants for complex brain diseases and/or brain-related traits with cell-type specificity [57]. Likewise, H3K4me3 PLAC in cortical brain nuclei identified microglia-specific enhancers/super-enhancers harboring Alzheimer’s risk variants, while psychiatric disease variants mostly affected neurons. Interestingly, most PLAC interactions linked disease risk variants to distal promoters and not to the closest active gene promoters [142]. Thereby, these techniques can be particularly useful for identifying epi/genetic loci spatially interacting with regulatory proteins in tissue homogenates [53,139,140], specific cell-types isolated using flow cytometry [55,57], and at single-cell level [58].

## 5. Conclusions

Given that non-coding genomic regions have been reported to be the hotspot of single-nucleotide or structural variants underlying complex traits, the integration of multi-omics approaches to profiling genomic architecture has identified functional roles of the non-coding causal risk variants in mediating complex diseases, particularly brain diseases. Gene-environment interactions mediated by activity-dependent changes at non-coding elements are found to be essential for normal brain development, whereas abnormal epigenetic changes at these regions during early-life may increase susceptibility to complex brain disorders. Moreover, human-specific genomic sequences that are under adaptive evolution include those non-coding elements that regulate genes important for cognitive functions but have also been found to harbor risk SNPs associated with psychiatric traits.

Notably, identifying disease risk genes based on their linear proximity or linkage disequilibrium has been insufficient. Accumulating evidence has shown that most risk variants enrich in distal regulatory sites and regulate gene expression through 3D chromatin loops. Moreover, 3D interactions have been found to outperform other paradigms in linking risk genes to disease risk loci. Additionally, germline and/or de novo risk variants are found to often disrupt transcription factor recognition sequences at distal gene enhancers or associate with differential histone modifications patterns in modifying cell-type specific transcriptome. Importantly, examining cell-type specific epigenetic changes using single-cell investigations is imperative to untangling biological complexity of polygenic traits in a heterogenous brain tissue and to allow unbiased discovery of rare cell-types or novel regulatory elements.

Overall, these findings illustrate the importance of employing chromatin profiling techniques in determining structures and functions of the chromatin environment. Moreover, these findings supported significant remodeling of chromatin states in driving altered gene expression networks underlying complex traits. Therefore, investigating open regulatory landscapes in cell- or cell-type specific manner using chromatin-profiling techniques is central to the quest of pinpointing epi/genetic targets associated with etiopathology of complex traits and diseases.

## Data Availability

This study did not report any data.

## Abbreviations

GWAS	Genome-wide association studies
LD	Linkage disequilibrium
SNPs	Single nucleotide polymorphisms
INDELs	Insertion/deletion polymorphisms
ENCODE	Encyclopedia of DNA Elements
TFs	Transcription factors
TSSs	Transcription start sites
CTCF	CCCTC-binding factor
TADs	Topologically associated domains
NPCs	Neural progenitor cells
FAC/NS	Fluorescence assisted cell/nuclei sorting
RNAPII	RNA polymerases II
DHSs	DNase I hypersensitive sites
DNase-seq	DNase I hypersensitive sites sequencing
MNase-seq	Micrococcal nuclease digestion of chromatin followed by sequencing
FAIRE-seq	Formaldehyde-assisted isolation of regulatory elements with sequencing
ATAC-seq	Assay for transposase-accessible chromatin coupled to sequencing
ChIP-seq	Chromatin immunoprecipitation with sequencing
CUT&RUN	Cleavage under targets and release using nuclease
DamID	DNA adenine methyltransferase (DAM)-identification
ChIA-PET	Chromatin interaction analysis by paired-end tag sequencing
PLAC-seq	Proximity ligation-assisted ChIP-Seq
